# Role of L-carnitine in protection against the cardiac oxidative stress induced by aspartame in Wistar albino rats

**DOI:** 10.1371/journal.pone.0204913

**Published:** 2018-11-07

**Authors:** Rasha A. Al-Eisa, Fawziah A. Al-Salmi, Reham Z. Hamza, Nahla S. El-Shenawy

**Affiliations:** 1 Biology Department, Faculty of Science, Taif University, Taif, Saudi Arabia; 2 Zoology Department, Faculty of Science, Zagazig University, Zagazig, Egypt; 3 Zoology Department, Faculty of Science, Suez Canal University, Ismailia, Egypt; National Institutes of Health, UNITED STATES

## Abstract

Aspartame (ASP) has been used as an alternative to sucrose for diabetics and obese people worldwide. Co-administration of L-carnitine (LC) with ASP has a protective effect against the liver and kidney toxicity induced of ASP. The goal of the investigation was to assess the enhancement of LC effect on the cardiac toxicity caused of ASP. The rats were divided into 6 groups: control with saline, LC (10 mg/kg), ASP (75 mg/kg), ASP (150 mg/kg), LC with 75 mg/kg of ASP, and LC with 150 mg/kg ASP. The antioxidants were determined by measuring the activities of myeloperoxidase, xanthine oxidase, superoxide dismutase, catalase, and glutathione peroxidase, and by assessing the levels of lipid peroxidation, total thiols, and glutathione. There was a significant elevation in LPO, in conjunction with a significant decline in the enzymatic antioxidants superoxide dismutase, catalase, and glutathione peroxidase and the non-enzymatic antioxidants glutathione and thiols. The cardiac myofibrils were found in a disarrayed pattern in ASP treated-animals as compared to the control rats. The animals treated with ASP-HD showed more than one apoptotic cell with a large tail and a small head, and the relaxed loops of the damaged DNA were extended to form a comet-shaped structure. These effects may be due to the excessive generation of reactive oxygen species by ASP, which reduces cardiac function. Co-administration of LC with ASP improved all of the above-mentioned parameters that were disrupted of ASP alone. This study evidences a sufficient originality in showing how LC plays a positive role against cardiac toxicity of ASP.

## Introduction

Aspartame (ASP) is metabolized in the gastrointestinal tract and is absorbed by circulating blood as 50% phenylalanine, 40% aspartic acid, and 10% methanol, which causes the toxicity of ASP as described in detail previously [1]. The USA and Europe have recommended the acceptable daily intake of ASP as 50 and 40 mg/kg of body weight, respectively [2].

Many studies have found that the mechanism of ASP toxicity in the liver, kidney, and brain depends on the level of elevation of oxidative stress [3,4,5]. Therefore, the side effects of ASP increase, due to an increase in reactive oxygen species (ROS) and an imbalance in the enzymatic/non-enzymatic antioxidant system, which causes dysfunction at the cellular level [5].

One of the studies showed that administration of ASP accounts for memory loss in some sensitive individuals [6]. Many authors have argued about the results regarding the ASP side effects as reported in detail previously [7; 8], and it is still widely used in foodstuffs as a healthy replacement, due to its lower number of calories. Moreover, Shahbaz et al. [9] suggested that ASP may contribute to cardiovascular pathologies that accompanies in human immunodeficiency virus (HIV).

However, the observations of the cardiac toxic effects of ASP are insufficient and only concern hypertension and electrocardiographic changes [10] or biochemical specifications [11]. Gudadhe et al. [12] found that ASP caused hypertrophy of myocytes, due to an increase in the dimeter of the myocytes of mice. Moreover, they found an elevated heart weight, due to an increase of semisolid matrix myocardium. Also, they explained that the reason for the hypertrophy of the cardiac myocytes could be to reimburse the loss of myocytes during an early period of the experiment.

Oxidative stress is highly correlated with ASP toxicity [13,14]. ASP induces reactive oxygen species formation and cytotoxicity, due to an imbalance in the antioxidants/oxidative system in hepatocytes [15]. It has been shown that free radicals may cause membrane destruction through the oxidative corruption of lipids, proteins, and DNA [16].

Lipid peroxidation is a marker of oxidative damage from ROS [17]. Targeting ROS-producing enzymes, such as myeloperoxidase (MPO, may also result in more selective ROS modulation under pathologic conditions. Furthermore, oxidative damage can be provoked by a decline in the activities of antioxidant enzymes, as they play a critical role in removing the free radicals associated with oxidative stress [18].

L-Carnitine (b-hydroxy-g-trimethyl-amino-butyric acid) is a pivotal component of mechanism of fatty acids transport across the mitochondrial membrane [19]. Carnitine simplify oxidation of long-chain fatty acids, modulates the ratio of CoA to CoA-SH, and is involved in peroxisomes and mitochondria residues removal. Carnitine also participates in branched chain amino acids metabolism and stabilizes the cellular membranes.

The current understanding of the toxic effects of ASP on the heart tissues of animals is limited; therefore, the present study was carried out to examine the hearts of rats, in order to clarify the possible alterations, due to the oxidative stress of cardiac myocytes, combined with the histological alteration of the myocardium, and to understand the effect of ASP. Moreover, the objective was to evaluate the ability of L-carnitine (LC) to decrease the toxicity in the presence of different doses of ASP.

## Materials and methods

### Chemicals

Aspartame (ASP) was supplied by Sigma-Aldrich chemical;USA, and the other chemicals were brought from Sisco Research Laboratory, Mumbai, India.

### Animal model and experimental design

Animal experiments were achieved after we obtained clearance from the Faculty of Pharmacy, Zagazig University Animal Ethical Committee (No. P22/2/2013). The experimental animals were healthy, inbreed adult male Wistar albino rats, weighing 200–250 g and were bring from Faculty Veterinary medicine–Zagazig University, Egypt. The animals were housed under the control condition (temperature 26.0 ± 2 °C with 12 h light and 12 h dark exposure) and were allowed to have basal diet and water *ad libitum* to minimize suffering. The specific criteria that used to monitor animal health were animal activity, health information were kept in accordance with organisation standard operating procedures. Signs of illness or injury and abnormal animal behaviour and conditions were recorded. Sick or injured animals were separated from other animals. Animal treatments were administered under supervision and dosages were in accordance with organization procedures and policies.

The Wistar rats were divided into six groups (n = 8) based on the administration treatment. The first group (control) was given saline. Second and third groups were treated with a lower dose of ASP (ASP-LD) and a higher dose of ASP (ASP-HD) (75 and 150 mg/Kg body weight, respectively) according to Iyyaswamy et al. [12]. Fourth group was treated with L-carnitine (LC; 10 mg/Kg) as recommended by Elshazly et al. [20]. The last two groups were treated with ASP-LD + LC and ASP-HD + LC, respectively at doses as mention above. All the animals were treated for 4 weeks orally successive days by gavage.

The animals were monitoring every day and the method of sacrificing of rats is using inhaled anesthetic with halothane.

### Heart homogenate preparation

Homogenates were prepared using phosphate buffer [1 mM/L Na_2_ EDTA, 10 mL of 500 mM/L butylated hydroxytoluene (BHT), pH 7.5]. The homogenates were centrifuged at 14K xg for 20 minutes and frozen at -20 C until analysis.

### Antioxidant evaluation

Myeloperoxidase (MPO) and xanthine oxidase (XO) activities were detected as described before by Suzuki et al. [21] and Litwack et al. [22], respectively. Levels of lipid peroxidation as MDA product besides the activities of superoxide dismutase (SOD), catalase (CAT) and glutathione peroxidase (GPx) were evaluated as antioxidant markers [23,24,25] and Hafeman et al. [26], respectively). Total thiols level was determined according to Hu [27] and glutathione (GSH) level was evaluated based on Beutler et al. [28].

### Histological examination

Heart tissue pieces were fixed in 10% formalin then it was removed by washing the samples with tap water overnight. The tissue was dehydrated using a series of alcohols, and were cleared in methyl benzoate and embedded in paraffin. Sections were cut using a microtome at the 6 μm thickness. The thin sections were stained with hematoxylin and eosin [29]. The slides were examined by light microscope and photographed by a digital camera.

### Comet assay

Pieces of the heart of control and treated groups were put into a cold solution consisted of Ca^2+^, Mg^2+^ free HBSS, 10% DMSO and 20 mM EDTA. The comet assay was evaluated as described by Endoh et al. [30]. The heart samples were more minced into finer pieces. Then, samples were filtered by100 μm nylon meshes and the cell suspensions were collected. All steps were carried in dark. An aliquot of 5 μl of cell suspension was mixed with 120 μl of 0.5% agarose at 37°C and placed on slides, pre-coated with 1.5% normal melting point agarose. The slides were placed in freshly prepared cold lysing solution (1% Triton X-100, 2.5 mM NaCl, 0.1 mM Na_2_ EDTA, 10 mM Tris with 10% DMSO, pH 10.0) overnight and then in a horizontal alkaline electrophoresis solution (0.3 M NaOH, 1 mM Na_2_EDTA, pH >13) at 4°C for 20 min.

The electrophoresis was performed at 25 V and 300 mA for 20 min. Then, the slides were washed twice for 5 min in buffer (0.4 M Tris HCl, pH 7.5), fixed for 5 min in absolute alcohol, air-dried, and stored at room temperature. Two slides from each animal were removed after lysis procedure to evaluate molecular weight DNA diffusion.

The DNA was stained with 50 μl of 20 μg/mL ethidium bromide. The slides were examined with a 40X objective lens with epi-illuminated fluorescence microscopy (Olympus-Bx60) attached to a camera and connected to an image analysis system (Comet II, Perspective Instruments, UK). The Comets were analyzed by a visual scoring method and computerized image analysis [31] to quantify DNA damage, tail length (TL), tail DNA (%) (TDNA) and tail moment (TM), 50–100 randomly selected cells are analyzed per sample.

## Statistical analysis

The SPSS 17.0 statistical software package program for Windows was used for statistical calculations. Data were given in the form of mean values ± standard error. Differences between groups were evaluated by one-way analysis of variance followed by post hoc Duncan test (*P* < 0.05).

## Results

The results are summarizes as the mean ± standard error (SE), and they show the effect of ASP on the oxidative/antioxidant criterion of the heart. There was a significant elevation in level of lipid peroxidation (LPO) and the activities of MPO and xanthine oxidase (XO). A significant decline in the activities of superoxide dismutase (SOD), catalase (CAT), and glutathione peroxidase (GPx) was observed, along with a decrease in the levels of glutathione (GSH) and protein thiols. The effect of ASP was dose-dependent (Table 1), compared to the control. These results confirmed the establishment of oxidative stress in the cardiomyocytes, associated with ASP in the animals.

**Table 1:** Oxidative/antioxidant parameters in heart of male rats treated with aspartame or/and L-carnitine.

Parameters	Control	LC	ASP- LD	ASP- HD	ASP- LD and LC	ASP- HD and LC
**MPO (nM/min/mL)**	7.90 ± 0.44	8.29 ± 0.56	14.90 ± 1.75^a^	13.53 ± 2.10 ^a^	11.30 ± 0.54	10.68 ± 0.70
**XO (U/g)**	11.64 ± 0.73	11.03 ± 0.51	19.99 ± 2.25 ^a^	22.16 ± 1.50 ^a^	12.76 ± 0.58 ^b^	12.82 ± 0.89 ^b^
**SOD (nM/g)**	22.96 ± 0.56	22.52 ± 1.14	9.50 ± 0.40 ^a^	8.99 ± 0.82 ^a^	14.57 ± 1.32 ^b^	15.13 ± 1.05 ^b^
**CAT (U/g)**	6.29 ± 0.21	6.65 ± 0.37	3.83 ± 0.19 ^a^	2.60 ± 0.18 ^a^	4.65 ± 0.40	5.03 ± 0.55
**GPx (M/g)**	20.34 ± 0.36	19.68 ± 0.78	9.07 ± 0.53 ^a^	7.03 ±0.50 ^a^	13.58 ±0.91 ^b^	13.21 ±1.11 ^b^
**Thiol (μM/g)**	7.14 ± 0.45	7.66 ± 0.42	4.59 ± 0.46 ^a^	3.66 ± 0.27 ^a^	6.78 ± 0.17 ^b^	6.36 ± 0.38 ^b^
**GSH (M/g)**	11.79 ± 0.58	12.78 ± 0.65	6.83 ± 0.57 ^a^	5.18 ± 0.49 ^a^	8.58 ± 0.58 ^b^	9.07 ± 0.60 ^b^
**MDA (nM/g)**	13.67 ± 2.26	15.78 ± 1.24	79.97 ± 5.98 ^a^	91.23 ± 2.14 ^a^	42.26 ± 2.93 ^b^	41.48 ± 4.65 ^b^

Values represent means ± SE; n = 8 for each treatment group. LC; L-carnitine, ASP- LD; the lower dose of aspartame, and ASP-HD; the higher dose of aspartame, MPO; Myeloperoxidase. XO; Xanthine oxidase, SOD; superoxide dismutase, CAT; catalase; GPx; glutathione peroxidase, GSH; reduced glutathione and MDA; malonildialdehyde.

^a^ significant difference as compared to control, and

^b^ significant difference (*P* ≤ 0.05). as compared to the corresponding group treated with ASP alone.

The obtained results indicated that the concentrations of LPO in the heart decreased significantly (p<0.05) in the group treated with LC and ASP, compared to the animals treated with ASP only. Moreover, administration of LC with ASP for 4 weeks significantly restored the enzymatic activities of SOD, CAT, and GPx to the levels of the control.

**Fig 1:**
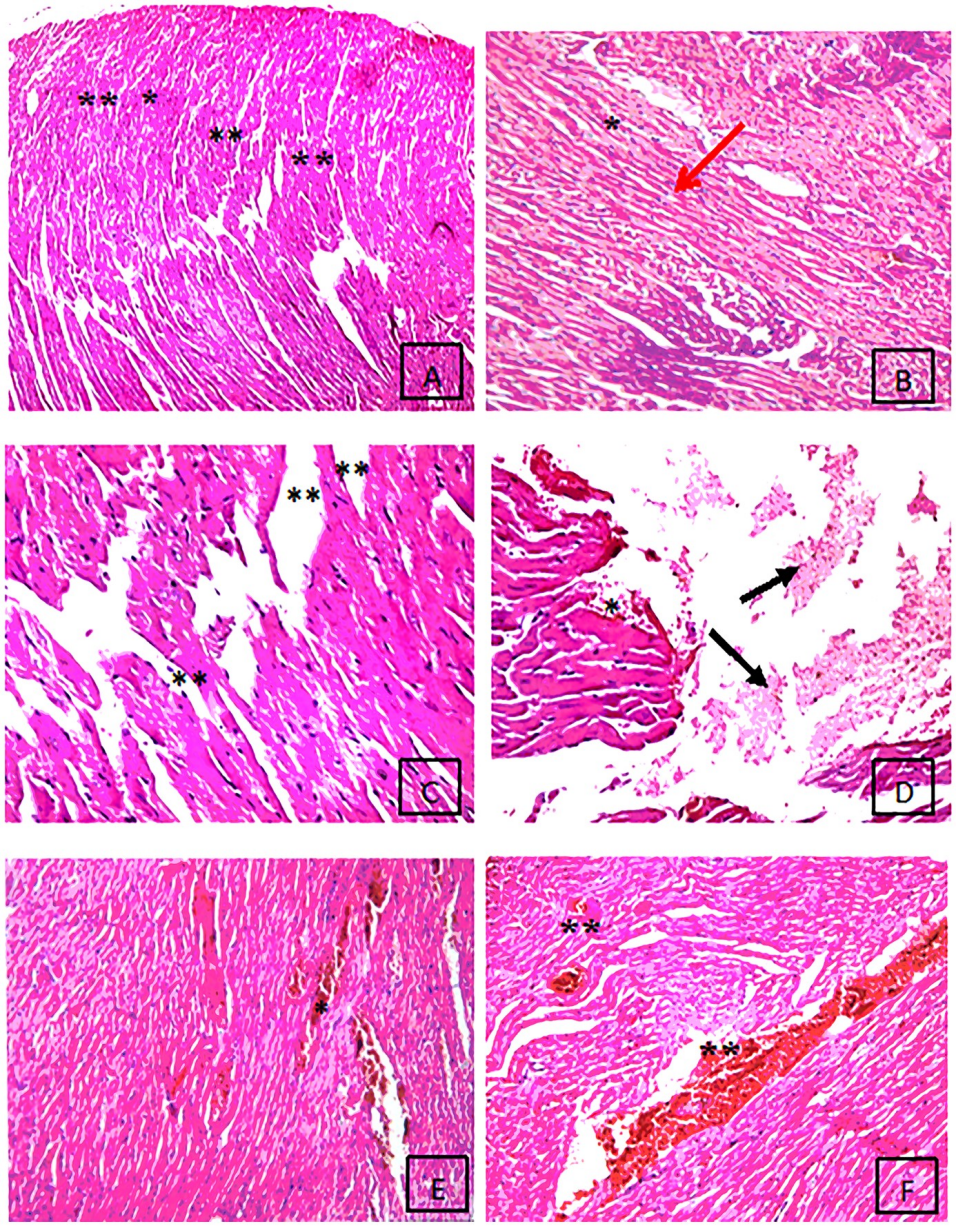
Histopathological slides of the heart stained with hematoxylin and eosin in the rat control group (A) where cardiac myocytes show normal appearance of cardiac muscle (**) formed of inter communicating muscle fibers in different directions (200X); the rat of L-carnitine (10 mg/Kg) group (B) show normal cardiac muscle (*) with normal sized nucleus (Red arrow) (400X); the animals of ASP-LD treated group (75 mg/kg) (C) show cardiac muscles have focal myocytes with ruptured muscle fibers (**) (400X); (D) the rats of ASP-HD treated group (150 mg/kg) show necrotic and atrophic muscle fibers (Black arrow) as well as areas of hemorrhage in between the cardiac muscle (*) (400X); (E) the group of ASP-LD and LC show partial restoration of cardiac muscles with mild congested area of muscle fibers (*) (400X); **(F)**: the rats of ASP-HD and LC group show restoration of cardiac muscles with moderate congested area (**) (400X).

Histological examination of the control group showed a normal cardiomyocyte pattern, characterized by individual, oval, and centrally-located nuclei with regularly-arranged cardiac myofibrils (Fig 1A). A similar structure was observed for the LC group. However, the nuclei of the cardiomyocytes in the ASP group were deformed in terms of their sizes and shapes (Fig 1B), and the cardiac myofibrils were found in a disarrayed pattern as compared to the control rats. The LC with ASP-LD group had fewer severe histological changes in the cardiac tissues compared to the ASP-LD animals (Fig 1).

**Fig 2:**
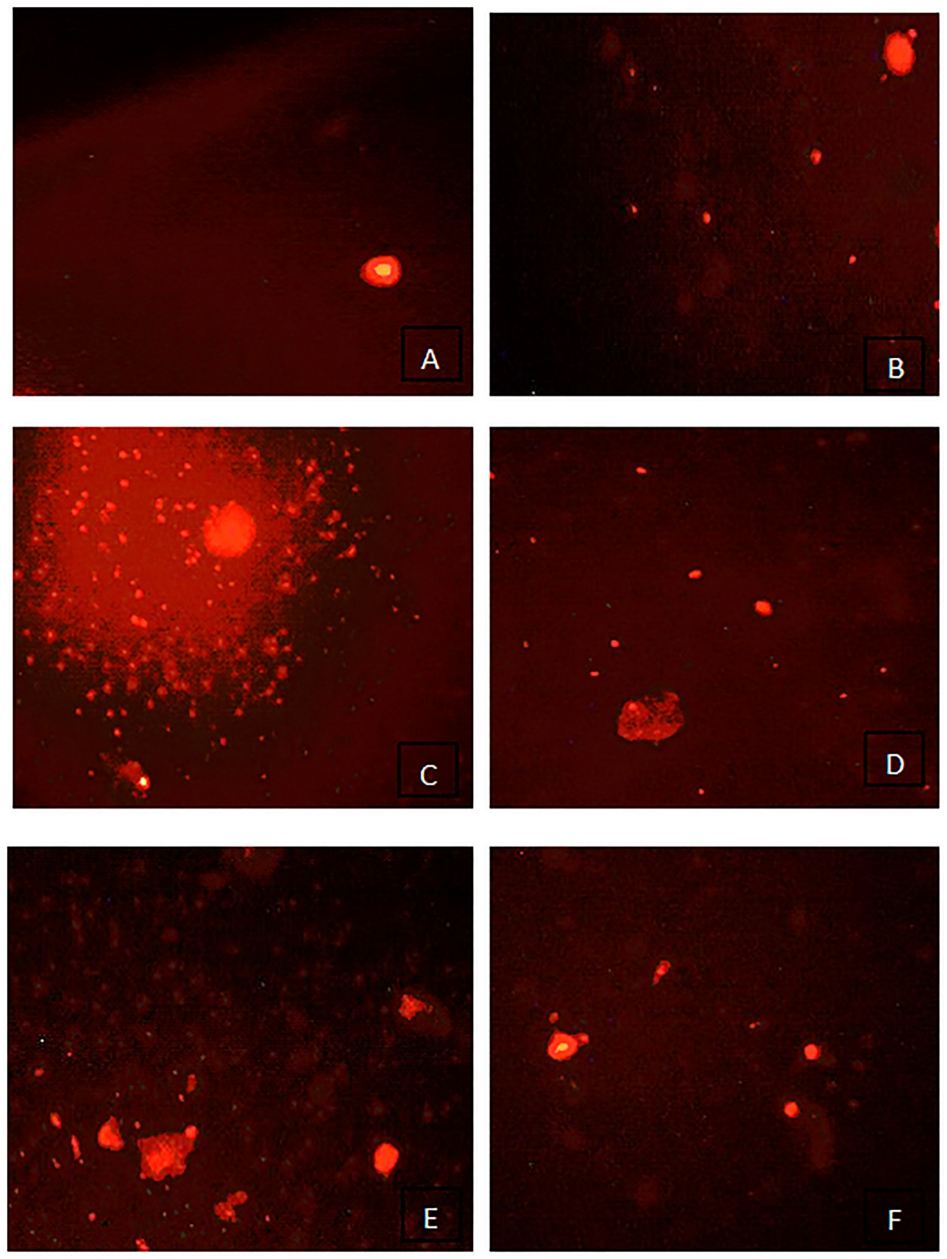
Comet images of cardiac myocytes from control rats (A) showing intact nuclei and normal round cell without tail, group LC (B) showing intact nuclei with undamaged DNA in a supercoiled state, group of ASP-LD (C) showing damaged DNA strand breaks which are revealed and damage nuclei as the cell contains a comet cell and with tail appear as hallow area, group of ASP-HD (D) showeing higher degree of damage with appearance of one large apoptotic cells with large tail and a very small head and the relaxed loops of damaged DNA, group of ASP-LD and LC (E) showing amelioration of the cells as recorded fewer parameters in the tail length and less of damaged DNA and tail and some with intact nuclei, and group of ASP-HD and LC (F) showing more percent of intact cells with undamaged DNA and less tail length and appeared some intact nuclei.

Table 2 summarizes the comet assay data, which are expressed as the tail moment and tail DNA % for the hearts exposed to different doses of ASP alone and ASP with LC. Cells exposed to a high dose of ASP exhibited a significant increase in single-strand breaks. Comet images of the cells derived from the heart are shown in Fig 2. The control and LC groups exhibited intact nuclei and normal, round cells without a tail (Fig 2A and 2B). The ASP-LD group showed damaged DNA with strand breaks and damaged nuclei, as the cells contained a head like a comet and a tail that appeared as a hollow area (Fig 2C). The animals treated with ASP-HD showed a higher degree of damage, in terms of the appearance of more than one apoptotic cell with a large tail and a small head, and the relaxed loops of the damaged DNA were extended to form a comet-shaped structure (Fig 2D). The ASP-LD + LC group showed amelioration of the cells, as evidenced by smaller parameters for the tail length and % of damaged DNA (Fig 2E). The ASP-HD + LC group had a higher percent of intact cells with undamaged DNA and fewer numbers of comet cells (Fig 2F).

**Table 2:** DNA damage of heart measured as comet percent tail damage and tail moment of male rats treated with aspartame or/and L-carnitine.

Groups	Tail Length (px)	% DNA in Tail	Tail Moment
Control	0.50 ± 0.08	0.46 ± 0.12	0.56 ± 0.02
LC	0.25 ± 0.06	0.32 ± 0.32	0.47 ± 0.74
ASP-LD	19.16 ± 1.74 ^a^	45.24 ± 2.56 ^a^	8.75 ± 0.35 ^a^
ASP-HD	22.81 ± 1.48 ^a^	52.35 ± 3.65 ^a^	13.63 ± 1.26 ^a^
ASP-LD + LC	4.29 ± 0.83 ^b^	10.11 ± 1.35 ^b^	4.86 ± 0.95 ^b^
ASP-HD + LC	5.07 ± 0.88 ^b^	11.41 ± 1.35 ^b^	8.35 ± 0.68 ^b^

Values represent means ± SE; n = 8 for each treatment group. LC; L-carnitine, ASP- LD; the lower dose of aspartame, and ASP-HD; the higher dose of aspartame.

^a^ significant difference as compared to control, and

^b^ significant difference (*P* ≤ 0.05). as compared to the corresponding group treated with ASP alone.

## Discussion

In this study, the effects of treatment with ASP alone or in combination with LC on the degree of oxidative/antioxidative stress as markers of cardiac dysfunction and the histological changes were examined.

The results indicated that MDA increased during ASP treatment, which can be taken as a direct confirmation of oxidative stress [32].

Hydroxyl radicals oxidize polyunsaturated fatty acids in biological membranes to induce the formation of lipid peroxides [33]. The results indicated that treatment with ASP increased lipid peroxidation in the heart. Other authors have shown that long-term ASP treatment elevates the levels of LPO in the brain [34], liver, and kidney tissues [14, 5,16]. The formation of LPO causes cellular membrane injury, allowing to membrane dysfunction, thus impairing the normal performance of the heart. This alteration may be due to methanol formation during ASP metabolism and formaldehyde release as part of methanol metabolism. This has been well confirmed by Parthasarathy et al. [35], who observed an increase in the LPO level in the lymphoid organs after methanol administration.

The antioxidative effect of GSH depends on its capacity to remove free radicals, to reduce peroxides, and to act as a co-substrate for the activity of GSH-dependent enzymes. The depletion of GSH increases the cell’s susceptibility to oxidative stress [36]. These changes could be due to the generation of free radicals by the methanol metabolites formed after ASP administration. GSH protects the cellular system against the toxic effects of LPO [37]. GSH reacts with superoxide, nitric oxide, hydroxyl radicals, and peroxynitrite radicals [38] to prevent their toxicities.

The present results demonstrated that ASP administration induces oxidative stress in the rat heart by altering the GSH level with the changes in LPO and other enzymatic antioxidants. The present findings suggest that long-term treatment with ASP can lead to the development of heart dysfunction.

SOD converts superoxide radicals to H_2_O_2_ [38]. However, NO competed with the SOD for O_2_^•−^ to form ONOO^−^ [39]. The present data indicate that the activity of SOD decreased significantly in the cardiomyocytes during the ASP treatment. The literature has shown that ASP chronic administration causes SOD activity to decline the liver and renal tissues [5,16], the spleen, thymus, lymph nodes, and bone marrow of rats [1]. The increased reactive oxygen species levels in the human heart are concerned with aortic valve stenosis [40].

During ASP treatment, the activity of CAT decreased. CAT is an enzyme that catalyzes the conversion of H_2_O_2_ to H_2_O and molecular oxygen [37]. Chronic administration of ASP increased CAT activity in the brain tissue of rats [38]. However, the increased production of ROS induced a decrease in CAT activity in the present study.

In the ASP-treated rats, the CAT and SOD activities decrease could be due to the elevated the free radicals production, as well as increased LPO levels and decreased concentrations of GSH. Thus, the GSH decreased levels resulted in the decrement of GPx activity. Elevation of LPO may act on the sulphhydryl groups present in the active sites of ATPases [41]. The membrane-bound enzymes are SH group-containing enzymes [42] that are sensitive to hydroperoxides and superoxide radicals [43]. Therefore, thiol modification (i.e. loss of a protein sulfhydryl group) has been recognized as a critical point for cytotoxicity [44].

All of these depleted parameters were restored by the administration of LC for 4 weeks. This fact can be explained by the ability of LC to remove the free radicals from circulation and to increase the expression of antioxidant enzymes, such as CAT, at the transcriptional level. Moreover, LC exhibits antioxidant effect by reducing metabolic stress and the use of LC has recently come into question in the treatment of many diseases [45].

The histological changes in the cardiac tissues of the ASP-treated rats indicated myocardial injury, as demonstrated by the deformation of the nuclei of the cardiomyocytes and the disarray or disorder of the cardiac myofibrils. These alternations are consistent with previous studies [46; 13].

The comet assay was used as a fast tool to rate the chemically-induced DNA damage. The ASP-LD and ASP-HD groups had a significantly increased percent tail DNA value, by approximately 100- and 113-fold, respectively, compared to the control. Therefore, ASP caused DNA damage, as detected by comet formation. These data are in parallel with those of Findikli and Turkoglu [47] who found the different sweeteners caused genotoxicity. DNA damage as an effect of ASP could be due to the generation of ROS from ASP metabolism, which causes DNA strand breaks and damage to the proteins responsible for DNA replication [48].

The administration of LC with ASP for 4 weeks improved the histological organization of heart cells by preventing the oxidative damage of the myocardium in the ASP-treated rats.

The LC protective effects against the oxidative stress induced by ASP could be contributed to its antioxidant defence in three different ways as previously described by Surai [49]; it acts as a free radical scavenging, inhibiting the enzymes responsible for free radical production and thus preventing the free radical formation. Maintaining electrontransport chain of mitochondria integrity especially in stress conditions, and by participating in the maintenance of optimal redox status of the cell by activating a range of antioxidant enzymes and non-enzymatic antioxidants, mainly via transcription factors, including Nrf2 and NF-κB. Finally, by activating an array of vitagenes network, responsible for the synthesis of protective molecules, including HSP, thioredoxin (Trx), sirtuins, etc., and providing additional protection in stress conditions. These are main mechanisms responsible for antioxidant action of LC and its derivatives.

Related to the disturbances of carnitine metabolism and myocardial function in experimental data: Carnitine is released from ischemic myocardium, and it’s concentration in the coronary sinus is proportional to the concentration of lactate [50,51]. These changes are reflected by a change in the ratio of free carnitine to carnitine esters in the heart.

LC supplementation beside causing an increment in ATP concentration was associated with a lower amount of toxic esters [52], Additionally, a declined in carnitine concentration in the heart was noticed in patients who died of myocardial infarction [53]. Thus all the previous experimental studies on the efficacy of LC in enhancing heart functions are completely in agreement with our finding in proving the role of LC in protection against the cardiac oxidative stress induced by aspartame.

## Conclusion

This experimental study on rats aimed to verify the protective role of LC against the cardiac toxicity of ASP. In this regard, such toxicity was investigated by assessing the heart antioxidant status, thus determining the activities of enzymes like MPO, XO, SOD, CAT and GPx as well as the levels of LPO, total thiols and GSH. Moreover, both histological examination and genotoxic evaluation (Comet assay) were performed at the cardiac level following treatment by gavage with two doses of ASP (75 and 150 mg/Kg body weight) alone or in association with LC (10 mg/Kg body weight). LC was shown to reverse the ASP-induced decrease of SOD, CAT and GPx activities, to reduce the levels of LPO and the activity of XO (increased by ASP) and to augment the levels of thiols and GSH that decreased by ASP. The results demonstrated that ASP induces the formation of free radicals, which are likely to cause oxidative stress and structural changes in cardiac tissue and impair cardiac function. The use of LC allows to protect the heart from both biological and structural injuries.

## Supporting information

S1 Fig Case for ASP (High group) with hepatomegaly and enlargerd heart, live showing highly oxidative stress in aspartame (High group) with hepatomegaly (Yellow arrow) and appearance of abnormal focal region in the liver with enlarged heart (Blue arrow) with appearant oxidative stress and more fats in different organs. (DOC)

S2 Fig Case for ASP (High group) with black colour heart and black veins which demonstrated the high damage in heart tissues by aspartame. (DOC)

S3 Fig Case for ASP (High group) with enlarged liver lobules (Blue arrow), heart with black veins which demonstrated the high damage in heart tissues by aspartame (Yellow and red arrows). (DOC)

S4 Fig Case for ASP (Low dose group) with darked oxidated liver and also heart (Blue arrow). (DOC)

S5 Fig Case for L-carnitine with normal and clear heart and liver (Blue arrow). (DOC)

S6 Fig Case for aspartame and L-carnitine with decreasing side effects of aspartame alone (Blue arrow). (DOC)

S1 Table Some biochemical data of antioxidant markers in heart tissues. (DOC)
