# Supplementary material for: Role of L-carnitine in protection against the cardiac oxidative stress induced by aspartame in Wistar albino rats
Source: PLoS One. 2018 Nov 7;13(11):e0204913. doi: 10.1371/journal.pone.0204913 (PMC6221268; doi:10.1371/journal.pone.0204913)

**Supporting information (S)**

**S1 Fig: Case for ASP (High group) with hepatomegaly and enlargerd heart, live showing highly oxidative stress in Aspartame (High group) with hepatomegaly (Yellow arrow) and appearance of abnormal focal region in the liver with enlarged heart (Blue arrow) with appearant oxidative stress and more fats in different organs**


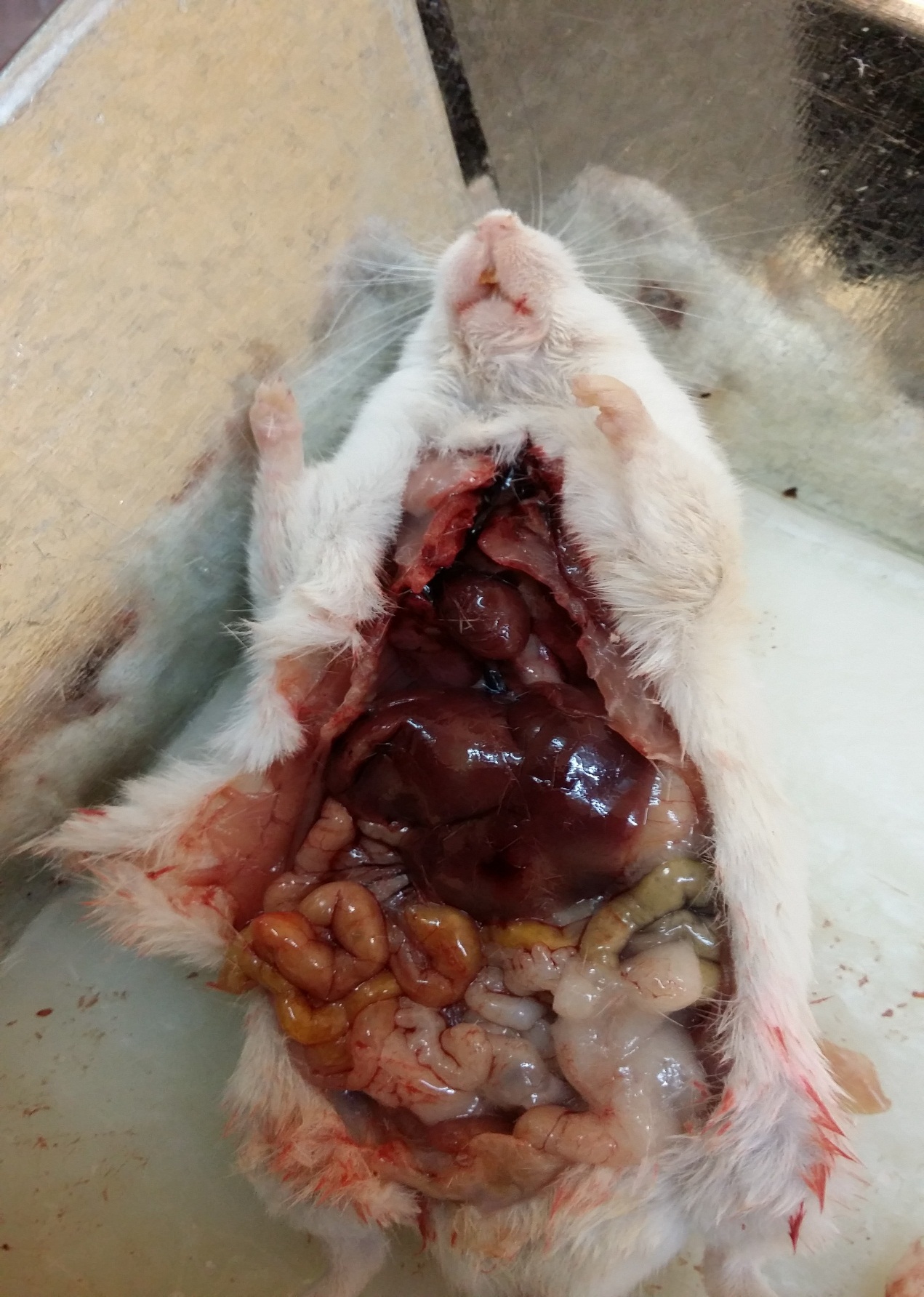

Supplement: S1 Fig — (DOC) [file pone.0204913.s001.doc]
