# Supplementary material for: Role of L-carnitine in protection against the cardiac oxidative stress induced by aspartame in Wistar albino rats
Source: PLoS One. 2018 Nov 7;13(11):e0204913. doi: 10.1371/journal.pone.0204913 (PMC6221268; doi:10.1371/journal.pone.0204913)

**S2 Fig: Case for ASP (High group) with black colour heart and black veins which demonstrated the high damage in heart tissues by aspartame**


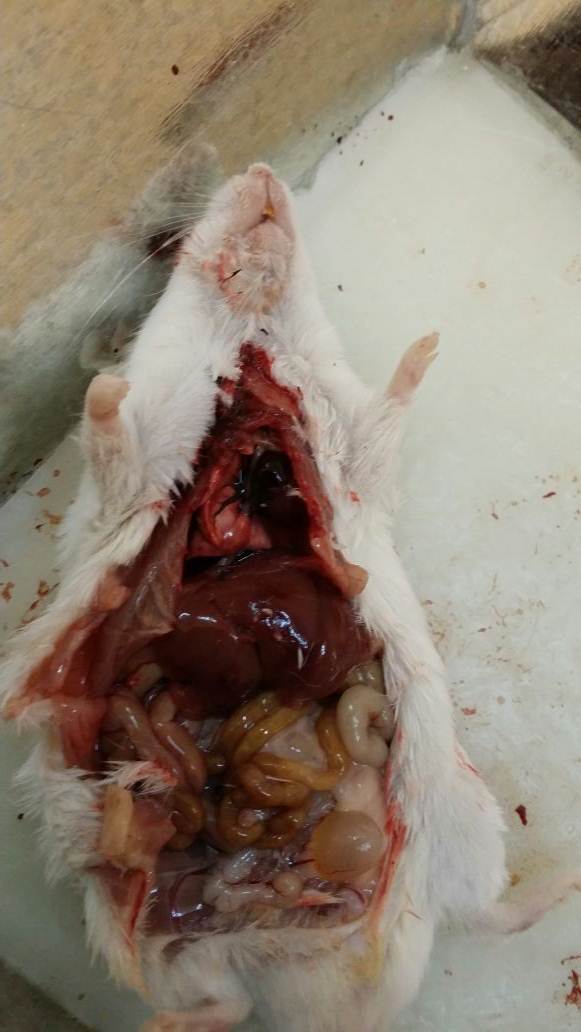

Supplement: S2 Fig — (DOC) [file pone.0204913.s002.doc]
