# Supplementary material for: Role of L-carnitine in protection against the cardiac oxidative stress induced by aspartame in Wistar albino rats
Source: PLoS One. 2018 Nov 7;13(11):e0204913. doi: 10.1371/journal.pone.0204913 (PMC6221268; doi:10.1371/journal.pone.0204913)

**S3 Fig: Case for ASP (High group) with enlarged liver lobules (Blue arrow) , heart with black veins which demonstrated the high damage in heart tissues by aspartame (Yellow and red arrows).**


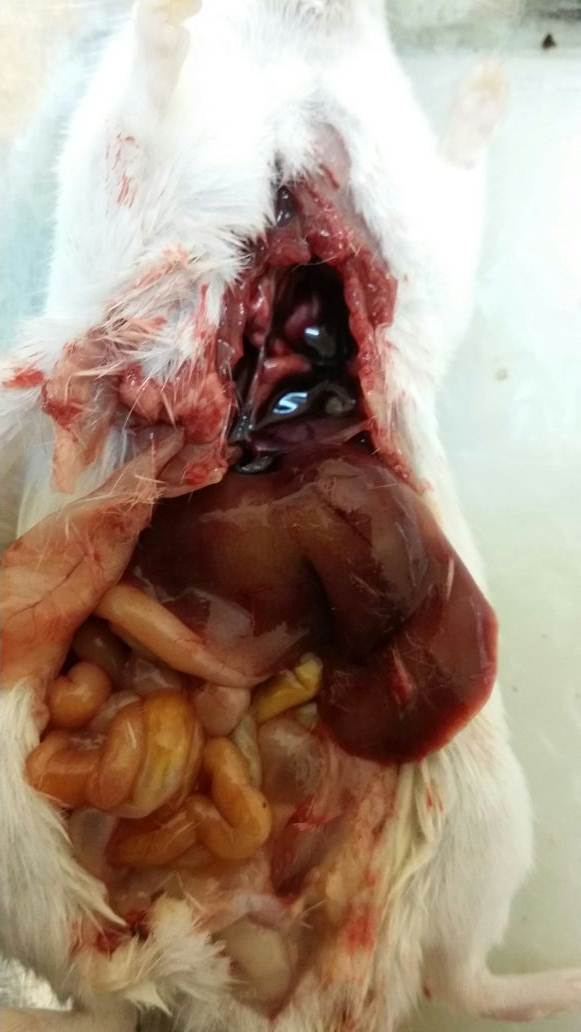

Supplement: S3 Fig — (DOC) [file pone.0204913.s003.doc]
