# Supplementary figures and images for: Role of L-carnitine in protection against the cardiac oxidative stress induced by aspartame in Wistar albino rats
Source: PLoS One. 2018 Nov 7;13(11):e0204913. doi: 10.1371/journal.pone.0204913 (PMC6221268; doi:10.1371/journal.pone.0204913)

**S4 Fig: Case for ASP (Low dose group) with darked oxidated liver and also heart (Blue arrow)**


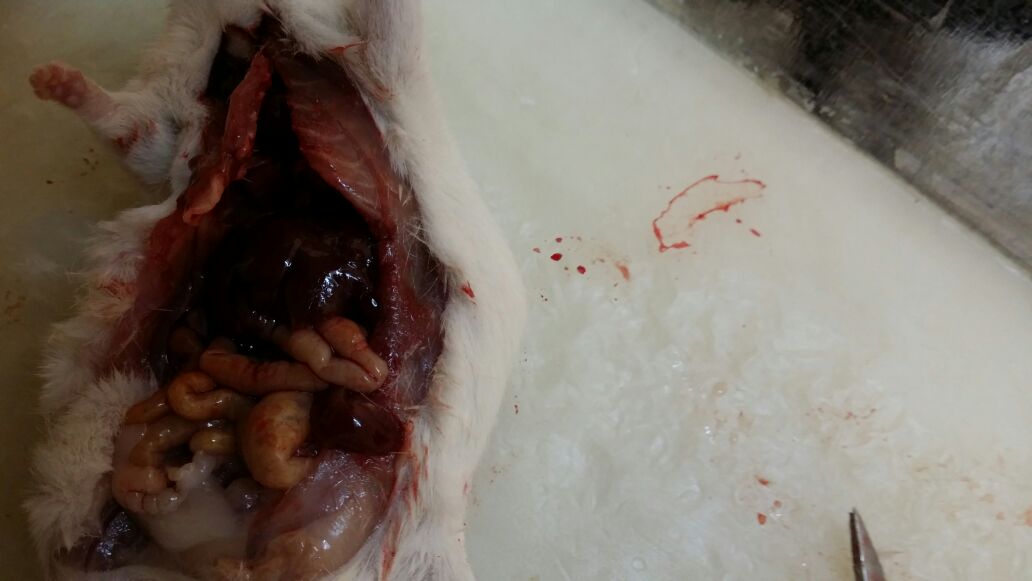

Supplement: S4 Fig — (DOC) [file pone.0204913.s004.doc]

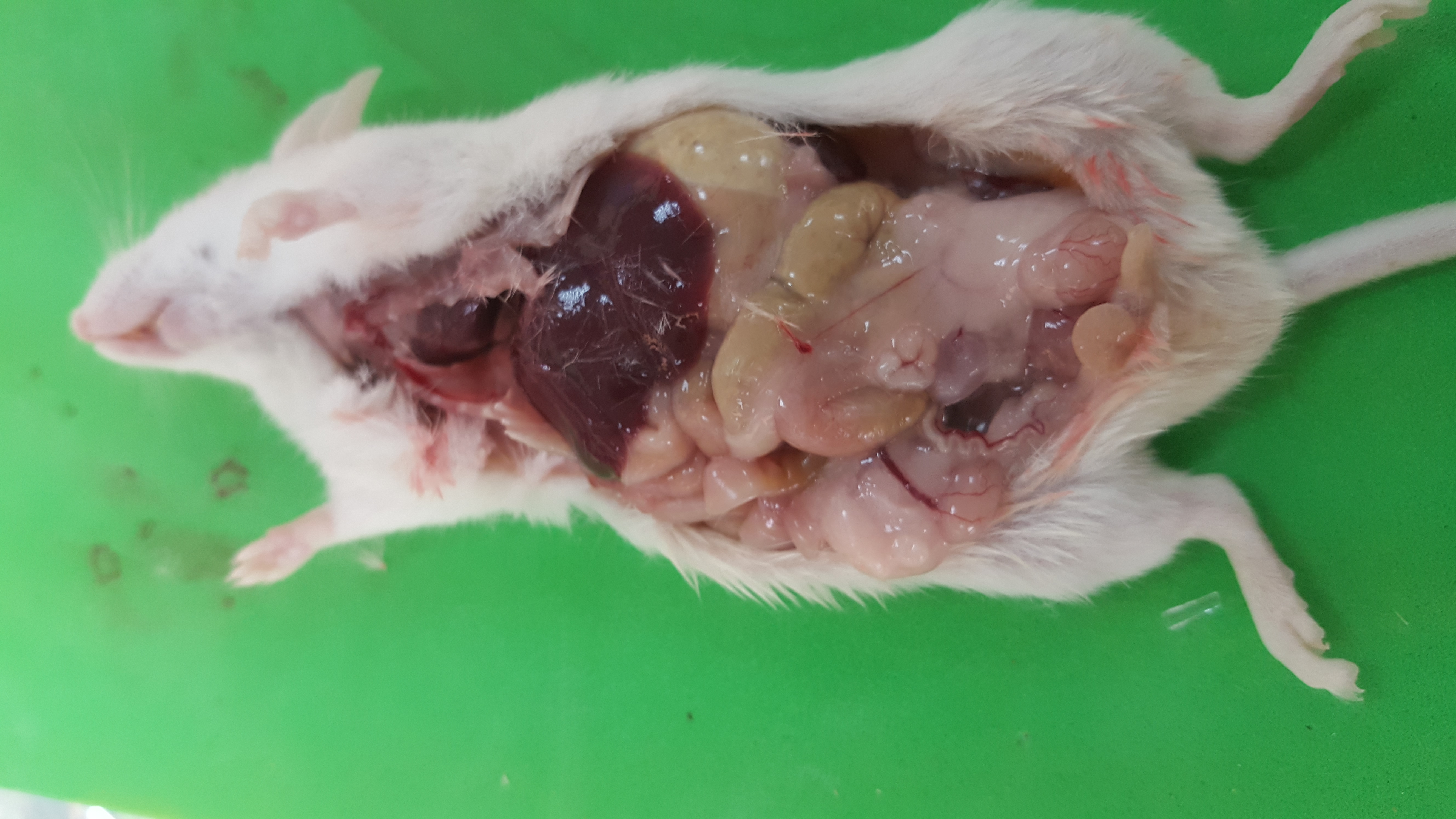
**S5 Fig: Case for L-Carnitine with normal and clear heart and liver (Blue arrow)**

Supplement: S5 Fig — (DOC) [file pone.0204913.s005.doc]
