# Supplementary material for: Role of L-carnitine in protection against the cardiac oxidative stress induced by aspartame in Wistar albino rats
Source: PLoS One. 2018 Nov 7;13(11):e0204913. doi: 10.1371/journal.pone.0204913 (PMC6221268; doi:10.1371/journal.pone.0204913)

**S6 Fig: Case for aspartame and L-Carnitine with decreasing side effects of aspartame alone (Blue arrow)**


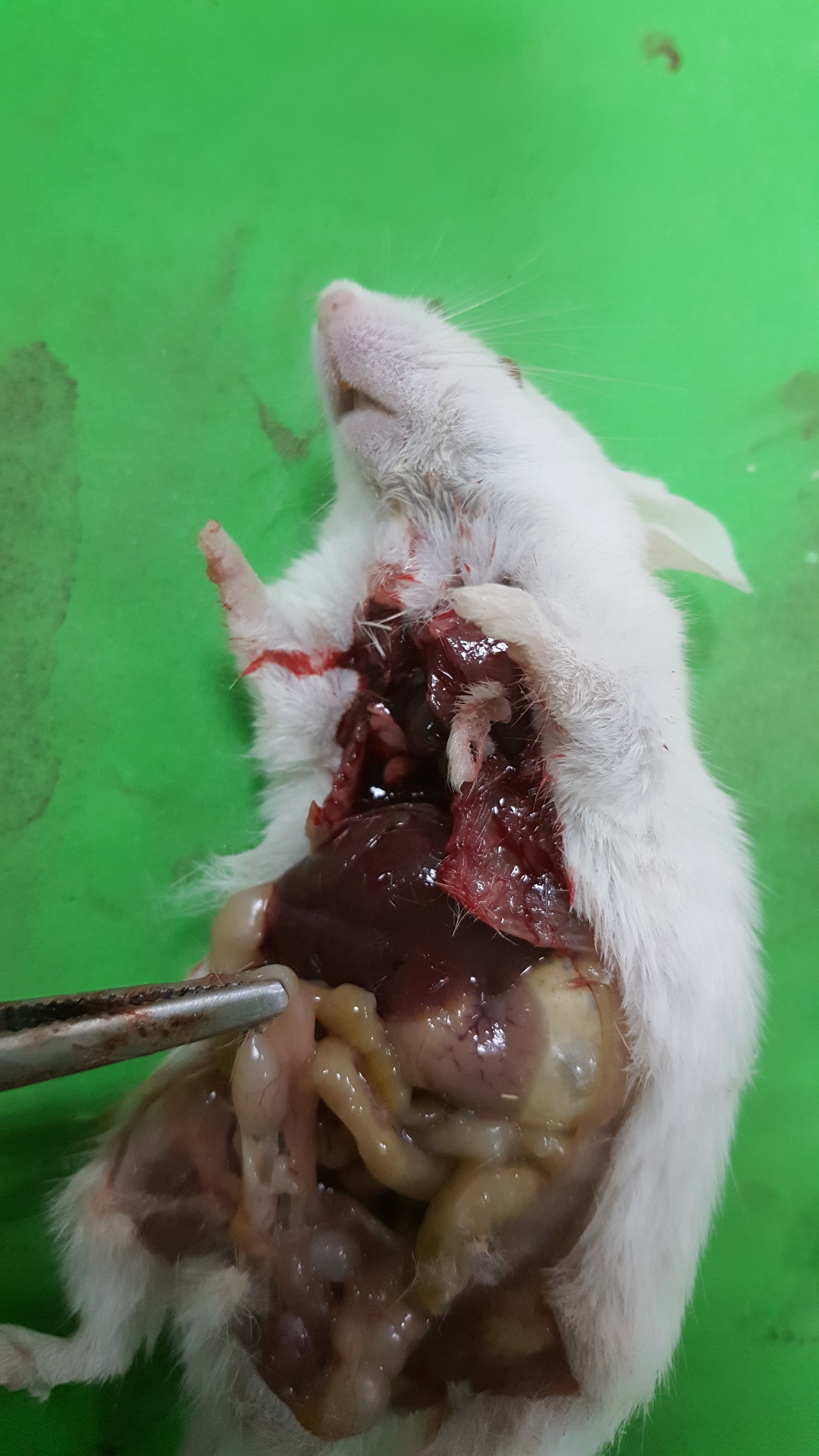

Supplement: S6 Fig — (DOC) [file pone.0204913.s006.doc]
