# Supplementary material for: Role of L-carnitine in protection against the cardiac oxidative stress induced by aspartame in Wistar albino rats
Source: PLoS One. 2018 Nov 7;13(11):e0204913. doi: 10.1371/journal.pone.0204913 (PMC6221268; doi:10.1371/journal.pone.0204913)
Supplement: S1 Table — (DOC) [file pone.0204913.s007.doc]

**S1 Tabel:** **some biochemical data of Antioxidant markers in heart tissues**

|  |  |  |  | **Antioxidant markers in Heart** | | | |
| --- | --- | --- | --- | --- | --- | --- | --- |
|  | **22.25** | **11.25** | **77.58** | | **99.25** | **40.25** | **50.24** |
| **MDA (nmol/g)** | **10.25** | **18.36** | **87.39** | | **87.95** | **43.25** | **31.25** |
|  | **11.24** | **17.58** | **57.58** | | **90.32** | **52.31** | **33.65** |
|  | **14.25** | **16.35** | **87.68** | | **87.25** | **41.25** | **37.58** |
|  | **10.36** | **15.36** | **89.64** | | **91.36** | **34.25** | **54.68** |
|  |  |  |  | |  |  |  |
| **SOD (nmol/g)** | **22.14** | **20.25** | **10.25** | | **7.25** | **11.25** | **18.25** |
|  | **23.35** | **19.5** | **9.25** | | **8.26** | **13.24** | **12.38** |
|  | **24.36** | **25.36** | **8.24** | | **11.24** | **18.68** | **13.46** |
|  | **21.25** | **24.25** | **9.25** | | **10.65** | **16.45** | **14.96** |
|  | **23.69** | **23.25** | **10.5** | | **7.56** | **13.25** | **16.59** |
|  |  |  |  | |  |  |  |
| **CAT (U/g)** | **6.25** | **6.57** | **3.25** | | **2.25** | **5.24** | **6.12** |
|  | **5.58** | **6.89** | **3.78** | | **3.24** | **4.32** | **5.87** |
|  | **6.32** | **7.59** | **3.65** | | **2.68** | **4.98** | **5.68** |
|  | **6.41** | **5.35** | **4.25** | | **2.47** | **3.25** | **4.25** |
|  | **6.87** | **6.87** | **4.21** | | **2.36** | **5.48** | **3.25** |
|  |  |  |  | |  |  |  |
| **GSH (mol/g)** | **11.25** | **11.65** | **7.25** | | **6.41** | **10.25** | **9.21** |
|  | **12.35** | **12.45** | **6.25** | | **6.32** | **8.25** | **8.65** |
|  | **10.25** | **13.86** | **5.24** | | **4.25** | **9.58** | **11.24** |
|  | **13.67** | **14.68** | **8.66** | | **4.25** | **7.25** | **7.58** |
|  | **11.45** | **11.25** | **6.74** | | **4.65** | **7.58** | **8.67** |
| **GPx (mol/g)** |  |  |  | |  |  |  |
|  | **20.35** | **20.34** | **10.25** | | **7.25** | **13.24** | **14.25** |
|  | **21.54** | **19.56** | **9.65** | | **6.32** | **15.48** | **10.36** |
|  | **19.67** | **18.57** | **9.63** | | **7.25** | **14.36** | **11.24** |
|  | **19.57** | **22.24** | **8.57** | | **8.65** | **10.25** | **16.57** |
|  | **20.58** | **17.68** | **7.25** | | **5.68** | **14.59** | **13.64** |
